# Association between obesity and calcium:phosphorus ratio in the habitual diets of adults in a city of Northeastern Brazil: an epidemiological study

**DOI:** 10.1186/1475-2891-12-90

**Published:** 2013-06-27

**Authors:** Danielle de Carvalho Pereira, Raquel Patrícia Ataíde Lima, Roberto Teixeira de Lima, Maria da Conceição Rodrigues Gonçalves, Liana Clébia Soares Lima de Morais, Sylvia do Carmo Castro Franceschini, Rosália Gouveia Filizola, Ronei Marcos de Moraes, Luiza Sonia Rios Asciutti, Maria José de Carvalho Costa

**Affiliations:** 1 Center for Health Sciences, Federal University of Paraiba, Castelo Branco, Joao Pessoa, PB 58059-900, Brazil; 2 Department of Nutrition, Center for Health Sciences, Federal University of Paraiba, Castelo Branco, Joao Pessoa, PB 58059-900, Brazil; 3 Department of Nutrition, Center for Health Sciences, Center for Biological and Health Sciences, Federal University of Viçosa, Viçosa, MG 36570-000, Brazil; 4 Faculty of Medical Sciences, Paraíba, Undergraduate Program in Nutrition, Centro, João Pessoa, PB 58010-740, Brazil

**Keywords:** Calcium: phosphorus Ratio, Obesity, Adult

## Abstract

**Background:**

Low calcium:phosphorus ratios (Ca:P ratio) in habitual diet have been observed worldwide, and it has been shown to be harmful to the bone health of the population. However, no study associating this ratio with obesity was found. Thus, considering that the intake of calcium and phosphorus will generate a ratio between them, which may be associated with obesity, this research seeks at evaluating the relation between obesity and the Ca:P ratio in the habitual diet of adults.

**Methods:**

Cross-sectional population-based epidemiological study with stratified and systematic sampling. The sample was composed of 506 adults, aged between 18 and 60 years, of both genders. Information on socioeconomic and demographic conditions was obtained through questionnaires completed during home visits, where anthropometric and dietary evaluations were also conducted.

**Results:**

In the habitual diet consumed by the study subjects, a Ca:P ratio above the median of 0.57 reduced the risk of central obesity based on waist-to-height ratio (WHtR) (OR: 0.61; 95% CI: 0.41 – 0.92). Habitual dietary intake of calcium (OR: 0.65; 95% CI: 0.43 – 0.97) and dairy products (OR: 0.56; 95% CI: 0.37 – 0.84) above the median value (485.4 mg and 0.9 servings, respectively) was found to be a protective factor related to central obesity based on WHtR.

**Conclusions:**

Values above the median for the Ca:P ratio found in the habitual diet were negatively associated with central obesity based on WHtR. In addition, calcium and dairy consumption were negatively associated with central obesity based on WHtR. Therefore, higher Ca:P ratios contributed to a lower prevalence of central obesity.

## Background

Obesity, which is prevalent in developed countries, has increased considerably worldwide. It is estimated that approximately 1.7 billion people worldwide are overweight, and of these, 310 million are affected by obesity [1]. According to data from the Household Budget Survey (Pesquisa de Orçamentos Familiares–POF 2008-2009), approximately half of all Brazilian adults are overweight, and 15% are obese [2].

Among the modifiable risk factors, dietary habits are the most closely related to the significant increase in the prevalence of overweight in the population [3]. Most studies that attempt to elucidate the mechanisms linked to dietary changes that may influence the energy balance are focused on macronutrients because they are energy nutrients whereas micronutrients, which have no caloric value, are less often studied [4].

Among the micronutrients, calcium plays a key role in bone health and is involved in vascular contraction and vasodilatation, muscle contraction, neural transmission and glandular secretion [5]. Cross-sectional and longitudinal studies have widely reported calcium’s effects on hypertension, metabolic syndrome, and obesity regulation [6-9]. The main mechanism suggested as responsible for calcium’s effect on obesity is the reduction in the intracellular calcium concentration of adipose cells, thereby preventing the storage of fat in these cells through inhibition of lipogenesis and stimulation of lipolysis and thus reducing weight gain [8]. According to recent research it can be observed that the adult population is not reaching the recommended daily calcium intake in various countries [10,11], including Brazil [12].

Phosphorus, which is essential for cellular signal transduction, mineral metabolism, and energy exchange [13], was also studied for its role in conjunction with calcium in bone formation and maintenance. However, although the consumption of this mineral is often two to three times what people need on a daily basis [14,15], studies relating phosphorus to chronic diseases are few. In these studies, phosphorus has been found to be inversely related to hypertension [16] and directly related to cardiovascular diseases [17,18], and there is no consensus regarding metabolic syndrome [8,19].

In the only study we found with an outcome of a relationship between phosphorus and obesity, a direct association was observed, with higher intakes of this nutrient associated with higher body mass index (BMI) and waist circumference (WC) [8].

Considering the calcium-phosphorus relationship in bone formation and their interaction during absorption and metabolism, the relationship between the consumption of these nutrients is an object of study and controversy in the literature [20], and a Ca:P ratio of 1.3:1.0 is suggested for maintaining bone health [15,21].

Reduced calcium intake and increased phosphorus intake, which are implied in a low Ca:P ratio, is a subject of concern worldwide [8,15,22,23] and surveys such as the POF 2002-2003 indicate similar changes in Brazil [24]. A higher Ca:P ratio in the diet is believed to prevent the reduction of bone mineral density (BMD) in human bone remodeling and to be an important factor in calcium balance [15,23,25].

However, no study was found that associated the Ca:P ratio in habitual diets with obesity. Thus, considering that this ratio may be associated with obesity, the aim of this population-based study was to determine the relationship between obesity and the Ca:P ratio in the habitual diets of adults in a city of Northeastern Brazil.

## Methods

### Study design and population

This study is linked to the research entitled "First diagnosis and intervention of food and nutritional status and the most prevalent non-communicable diseases of the population from the city of João Pessoa-PB/Northeastern Brazil (DISANDNT I/JP)". The study was conducted from July 2008 to January 2010.

This cross-sectional, population-based epidemiological study was conducted using a stratified and systematic sample that covered the city’s population using the R Development Team software [26]. During the initial research, information on individuals of all ages was collected; however, in this study, only the adult population was considered. Data collection was performed by properly trained staff, with the assistance of master’s candidates from the Post-Graduate Program in Nutritional Sciences (PPGCN) at the Federal University of Paraíba (UFPB) as coordinators and implementers of data collection together with other team members, and was conducted during home visits. Complete information about the sample definition and data collection is provided in a previously published study developed by part of the research team [27].

The adult population included individuals between 18 and 60 years of age of both genders and various socioeconomic conditions. A total of 1132 adults represented 55.76% of the individuals of all ages in these households (n = 2030). If there were two or more adults in one household (only one adult from each household was designated to participate in the study), the participant was randomly selected; thus, the number of selected adults was 527. Adults who used calcium, phosphorus or polymineral supplements and pregnant women were excluded. Thus, the final sample number was 506 adults. Of these, 410 subjects provided complete anthropometric and dietary data, leading to a participation rate of 81.03%.

The sample can be considered as representative of the Brazilian population, since the percentage of adults found in households chosen randomly in relation to the number of inhabitants in these households (55.76%), is similar to the one found for the adult Brazilian population (56.22%) [28].

Individuals who agreed to participate in all stages of the study were asked to read and sign the "Free Informed Consent Form". This study was approved by the Ethics Research Committee for the Center for Health Sciences (CCS) at the Federal University of Paraíba under protocol number 0493.

### Socioeconomic assessments

Data on per capita income were obtained by dividing the total proceeds received by the family by the number of components of that family. This data were classified according to the median found in the study: lower than the median and greater than or equal to the median. Educational level was established according to the classifications of less than or equal to high school and higher than high school.

### Anthropometric assessments

Weight, height and waist circumference of participants were measured. Each procedure was performed in triplicate, and the mean values were used.

Obesity was assessed by three parameters: body mass index (BMI), which defined the overall obesity when ≥ 30 kg/m^2^[29]; waist circumference (WC), which in men defined as central obesity when > 102 cm and in women when > 88 cm [30]; and waist-to-height ratio (WHtR), using the cutoff points from a previous study conducted in two groups of adults, a group of individuals with one or less cardiovascular risk factor and the other with more than 1 cardiovascular risk factor from District IV of the city of João Pessoa, which were 0,56 for both genders [31]. This parameter was chosen because it is reported in the literature as a good indicator of central obesity [32,33], but also by the lack of research relating it with the Ca:P ratio in habitual diets.

### Practice regular physical activity and dietary assessments

Data on physical activity were collected based on the following issues: practice physical activity, number of times per week and the period of time in minutes. It was considered as practicing regular physical activity the individuals who performed at least 150 minutes of physical activity per week, as recommended by the American College of Sports Medicine [34].

The food survey was conducted using the "Frequency Questionnaire Quantitative Food Consumption" (FQQFC) validated from three 24-hour recalls applied at various time intervals for the female population of the city of João Pessoa, PB, Brazil. The survey was conducted in partnership with the Public Health School, University of Sao Paulo, and the Post-Graduate Program in Nutritional Sciences, Federal University of Paraíba [35,36]. The FQQFC is best described in a previously published article [27].

Quantification of habitual dietary calcium and phosphorus intake was performed with the aid of the Dietsys software [37] (version 3.0).

For the analyses of nutrient requirements, recommendations from the dietary reference intake (DRI) were adopted according to the age group studied for calcium and phosphorus using the Estimated Average Requirement (EAR), which for calcium is 1.100 mg/day for individuals aged 18 years, 800 mg/day for individuals 19 to 50 years and men 51 to 60 years, and 1000 mg/day for women aged 51 to 60 years [38]. For phosphorus, the needs are of 1055 mg/day for individuals aged 18 and 589 mg/day for individuals 19-60 years [5].

Nutrients were energy-adjusted by the method of nutrient density, per 1000 kcal, and were also used in the analyzes for correcting the effect of energy intake on consumption of nutrient.

The consumption of major food groups and sources of calcium and phosphorus were also evaluated. Dairy products were considered the main food sources of calcium whose recommendation for adults is 2 to 3 servings daily [39,40]. It was considered as one portion of dairy the equivalent to 200 mL of whole milk or skim milk, 200 mL of yoghurt or 40 g of cheese. Regarding the group of meat generally, recognized food as major sources of phosphorus, its recommendation is 1-2 servings daily, considering 1 serving the equivalent to 80 g of red meat or 100 g of chicken or fish [39,40].

### Statistical analysis

Statistical analyses took into account the sampling strategy. Initially, an analysis of sample characteristics was conducted through descriptive statistics represented by single frequency, using measures of position such as central tendency and dispersion (mean, median, and standard deviation). Data were assessed for normality using the Lilliefors normality test, which is a derivative of the Kolmogorov-Smirnov test [41]. Because of the non-normality of the data, socioeconomic variables and practice physical activity were investigated for association with habitual food intake and anthropometric variables, using the Wilcoxon-Mann–Whitney test [41]. Also because of the non-normality of the data, the relationship between obesity parameters and the Ca:P ratio in the habitual diet and other consumption variables was investigated using the Spearman rank correlation. We then calculated odds ratio crude (OR) and confidence intervals at 95% between anthropometric measures and nutrients and habitual food consumption. We also calculated the adjusted OR using multiple regression in four models of sequential adjustment between anthropometric measures and nutrients [41]. All statistical analyses were performed using the R Development Team software [26]. A value of p < 0.05 was considered to be significant.

## Results

Data regarding general characteristics, socioeconomic status, habitual food consumption and anthropometric measures of adults of the DISANDNT I/JP research are shown in Table 1.

**Table 1:** General characteristics of adults of DISANDNT I/JP (2008-2010)

			**Total (Men and women)**				**Men**				**Women**			
			**Mean (± SD)**		**Range**	**N (%)**	**Mean (± SD)**		**Range**	**N (%)**	**Mean (± SD)**		**Range**	**N (%)**
General and socioeconomic characteristics														
	Gender					506 (100)				105 (20.75)				401 (79.25)
	Age (years)		37.71 (± 11.96)		41.00		36.13 (± 12.62)		41.00		38.20 (± 11.77)		41.00	
	Regular physical activity^1^													
		Yes				89 (17.59)				33 (31.43)				56 (13.97)
		No				417 (82.41)				72 (68.57)				345 (86.03)
	Educational level ^2^													
		≤ High school				392 (77.47)				73 (69.52)				319 (79.55)
		> High school				114 (22.53)				32 (30.48)				82 (20.45)
	Per capita income^3^													
		< Median				242 (49.90)				39 (39.80)				203 (52.45)
		≥ Median				243 (50.10)				59 (60.20)				184 (47.55)
Habitual dietary intake of nutrients														
	Ca:P ratio (mg:mg)		0.58 (± 0.17)		0.91		0.59 (± 0.17)		0.72		0.58 (± 0.17)		0.91	
	Ca (mg)		552.09 (± 325.24)		2603.40		670.28 (± 423.19)		2570.40		521.24 (± 287.24)		1624.10	
	P (mg)		927.49 (± 401.66)		2589.90		1086.32 (± 460.92)		2398.70		886.03 (± 374.50)		2589.90	
Habitual dietary consumption of food and food groups^4^														
	Dairy products (servings)		1.14 (± 0.98)		7.09		1.40 (± 1.23)		7.09		1.08 (± 0.89)		4.15	
		Whole milk (mL)	116.94 (± 140.72)		803.57		160.67 (± 181.37)		803.57		105.65 (± 126.04)		610.00	
		Skim milk (mL)	20.67 (± 71.22)		450.00		15.14 (± 60.88)		450.00		22.10 (± 73.67)		450.00	
		Yogurt (g)	34.81 (± 69.06)		990.00		40.25 (± 115.10)		990.00		33.41 (± 51.01)		495.00	
		Cheese (g)	11.21 (± 12.03)		64.67		12.60 (± 12.23)		52.86		10.85 (± 11.98)		64.67	
		Whole milk (servings)	0.58 (± 0.70)		4.02		0.80 (± 0.91)		4.02		0.53 (± 0.63)		3.05	
		Skim milk (servings)	0.10 (± 0.36)		2.25		0.08 (± 0.30)		2.25		0.11 (± 0.37)		2.25	
		Yogurt (servings)	0.17 (± 0.35)		4.95		0.20 (± 0.58)		4.95		0.17 (± 0.26)		2.48	
		Cheese (servings)	0.28 (± 0.30)		1.62		0.31 (± 0.31)		1.32		0.27 (± 0.30)		1.62	
	Meats (servings)		1.79 (± 1.23)		13.37		1.91 (± 1.18)		6.89		1.76 (± 1.24)		13.34	
		Red meat (g)	89.44 (± 81.76)		1044.29		92.47 (± 77.79)		424.61		88.66 (± 82.85)		1044.29	
		Poultry (g)	50.34 (± 53.50)		664.29		55.38 (± 53.76)		381.43		49.03 (± 53.44)		664.29	
		Fish (g)	22.35 (± 41.70)		712.00		25.83 (± 34.67)		200		21.45 (± 43.34)		712.00	
		Red meat (servings)	1.06 (± 0.97)		12.43		1.10 (± 0.93)		5.05		1.06 (± 0.99)		12.43	
		Poultry (servings)	0.50 (± 0.54)		6.64		0.55 (± 0.54)		3.81		0.49 (± 0.53)		6.64	
		Fish (servings)	0.22 (± 0.42)		3.00		0.26 (± 0.35)		2.00		0.21 (± 0.43)		7.12	
Anthropometric characteristics														
	Weight (kg)		66.47 (± 14.96)		106.00		72.91 (± 16.69)		106.00		64.78 (± 14.01)		85.70	
	Height (m)		1.60 (± 0.09)		0.81		1.70 (± 0.10)		0.71		1.57 (± 0.07)		0.79	
	BMI (kg/m^2^)		26.04 (± 5.42)		36.26		25.22 (± 4.71)		24.08		26.26 (± 5.58)		36.26	
	BMI classification^5^													
		Underweight				28 (5.57)				8 (7.69)				20 (5.01)
		Normal weight				201 (39.96)				46 (44.23)				155 (38.85)
		Overweight				163 (32.41)				35 (33.65)				128 (32.08)
		Obesity				111 (22.07)				15 (14.42)				96 (24.06)
	WC (cm)		84.86 (± 13.31)		76.00		87.45 (± 12.61)		68.30		84.19 (± 13.41)		76.00	
	Central obesity based on WC^6^					159 (31.67)				11 (10.68)				148 (37.09)
	WHtR (cm:cm)		0.53 (± 0.09)		0.43		0.51 (± 0.07)		0.41		0.54 (± 0.09)		0.42	
	Central obesity based on WHtR^7^					178 (35.53)				24 (23.53)				154 (38.60)

^1^ at least 150 minutes of physical activity per week [34]; ^2^ ≤ high school, corresponding to 12 or fewer years of schooling and > high school, corresponding to more than 12 years of schooling; ^3^ median of per capita income R$ 325.00 or US$ 580.29; ^4^ food consumption – servings (BRASIL. Ministério da Saúde. Guia Alimentar para a população brasileira, 2006 [39]/USDA. U.S. Department of Agriculture. Dietary Guidelines for Americans, 2010) [40]; ^5^ WHO (1998); ^6^ NCEP-ATPIII (2002); ^7^ central obesity WHtR ≥ 0.56 seconds VIEIRA (2009).

Abbreviations: *SD* Standard deviation, *Ca:P ratio* Calcium:phosphorus ratio, *Ca* Calcium, *P* Phosphorus, *BMI* Body mass index, *WC* Waist circumference, *WHtR* Waist-to-height ratio.

### General and socioeconomic characteristics

The total sample (n = 506) consisted predominantly of women. Less than one-third of the participants practiced regular physical activity, with higher rates among men than women. An educational level above the school also found in less than 1/3 of the sample, and the ratio was also higher among men. Data on per capita income were provided by 485 study participants, with just over half of the population being above the median.

### Ca:P ratio, Ca and P in habitual diets

A dietary analysis was performed on 410 of the total sample of 506 individuals. Some participants did not complete or did not respond to the FQQFC, and others were excluded for reporting an energy consumption that was considered implausible (energy value <700 kcal or >5000 kcal). The mean Ca:P ratio from the habitual diet was 0.58, that is, a ratio of approximately 1:1.72. The median for the same ratio was 0.57 or approximately 1:1.75. Average dietary calcium intake was lower than the desired value; 83.95% of the population did not meet the DRI for this nutrient, and 39.77% consumed less half of the requirement. However, dietary intake of phosphorus was greater than the DRI in 81.63% of the sample, 20.93% of whom had 200% higher consumption for this nutrient.

### Foods and food groups rich in Ca and P

The average habitual consumption of dairy products (1.14 servings, corresponding to 228 mL of milk or yogurt or 45.6 g of cheese) was well below recommended levels, although 98.31% of the sample reported consuming at least one type of food from this group. In this group, whole milk was the most commonly consumed food, but with an average consumption of less than one serving per day. A small percentage of individuals reported consuming skim milk (13.04%), thereby contributing little to the overall average consumption of this food. Consumption of yogurt and cheese was reported by most participants, although infrequently, resulting in low average daily intake. In terms of meats, only one individual did not consume any product from this group, and daily average consumption was 1.79 servings, corresponding to 150.4 g of red meat or 179 g of poultry or fish, which is in accordance with the recommendation of 1 to 2 servings per day. Red meat was the most commonly consumed meat, followed by poultry and, to a lesser degree, fish, the consumption of which was also reported by a large number of individuals, although infrequently.

### Anthropometric characteristics

More than half of the total sample (54.48%) had some level of overweight, and of these, 22.07% were obese. Regarding WC and WHtR, central obesity was identified in approximately one-third of the sample. For all obesity diagnostic parameters, women had higher proportions than men (p < 0,001).

### Socioeconomic characteristics x food consumption and obesity

Higher education level (> high school, corresponding to more than 12 years of schooling) and per capita income greater than or equal to median (≥ R$ 325.00, corresponding to ≥ US$ 580.29), and practice regular physical activity were significantly related to higher Ca:P ratio and intake of calcium and phosphorus (crude and energy-adjusted), and dairy products. Neither the consumption of meats nor the obesity diagnostic parameters were related to these factors, except for WHtR, which was inversely and significantly related to educational level (Table 2).

**Table 2:** Mean and median of Ca:P ratio, nutrients, food groups in the habitual diet and obesity diagnostic parameters according to education level, per capita income and practice of regular physical activity of adults of DISANDNT I/JP (2008-2010)

**Variables**	**Educational level **^**1**^					**Per capita income **^**2**^					**Regular physical activity **^**3**^				
	**≤ High school**		**> High school**			**< Median**		**≥ Median**			**Yes**		**No**		
	**Mean**	**Median**	**Mean**	**Median**	**p-value**	**Mean**	**Median**	**Mean**	**Median**	**p-value**	**Mean**	**Median**	**Mean**	**Median**	**p-value**
Ca:P ratio (mg:mg)	0.57	0.56	0.63	0.64	0.0006	0.55	0.53	0.61	0.61	0.0001	0.63	0.64	0.57	0.56	0.0110
Calcium* (mg)	522.19	453.10	653.39	529.20	0.0006	496.70	426.20	607.47	556.80	0.0002	609.01	569.05	540.26	475.70	0.0149
Phosphorus* (mg)	897.75	840.60	1028.2	906.10	0.0371	887.24	820.80	967.94	910.25	0.0411	957.56	904.00	921.24	840.65	0.1929
Dairy products (servings)^4^	1.01	0.79	1.61	1.31	0.0001	0.91	0.74	1.38	1.10	0.0001	1.38	1.21	1.09	0.83	0.0033
Meats (servings)^3^	1.82	1.53	1.68	1.31	0.067	1.89	1.58	1.69	1.41	0.0994	1.66	1.47	1.82	1.51	0.2322
BMI (kg/m^2^)	26.16	25.54	25.59	25.04	0.2472	26.16	25.20	25.98	25.86	0.8582	26.09	25.89	26.01	25.30	0.8024
WC (cm)	84.99	84.00	84.55	83.00	0.5845	84.25	83.00	85.46	85.00	0.1554	85.45	84.70	84.73	83.40	0.4801
WHtR (cm:cm)	0.54	0.51	0.51	0.50	0.0126	0.53	0.53	0.53	0.53	0.8506	0.53	0.52	0.53	0.53	0.4870

^1^ ≤ high school, corresponding to 12 or fewer years of schooling and > high school, corresponding to more than 12 years of schooling; ^2^ median of per capita income R$ 325.00 or US$ 580.29; ^3^ at least 150 minutes of physical activity per week [34]; ^4^ food consumption - servings (BRASIL. Ministério da Saúde. Guia Alimentar para a população brasileira, 2006 [39]/USDA. U.S. Department of Agriculture. Dietary Guidelines for Americans, 2010) [40].

* Calcium and phosphorus - after adjustment for energy remained the same significant relationships.

Abbreviations: *Ca:P ratio* Calcium:phosphorus ratio, *BMI* Body mass index, *WC* Waist circumference, *WHtR* Waist-to-height ratio.

### Habitual food intake and its relationship with obesity diagnostic parameters

Correlation tests revealed a significant inverse association between Ca:P ratio in the habitual diet and WC and WHtR values (Table 3).

**Table 3:** Correlation between Ca:P ratio in the habitual diet and obesity diagnostic parameters of adults of DISANDNT I/JP (2008-2010)

	**BMI (kg/m**^**2**^**)**		**WC (cm)**		**WHtR (cm:cm)**	
	**r**	**p**	**r**	**p**	**r**	**p**
Ca:P ratio (mg:mg)	-0.36	0.45	-0.11	0.03*	-0.11	0.02*

Abbreviations: *SD* Standard deviation, *Ca:P ratio* Calcium:phosphorus ratio, *BMI* Body mass index, *WC* Waist circumference, *WHtR* Waist-to-height ratio.

The medians of the Ca:P ratio, nutrients, foods and food groups studied were associated with the obesity diagnostic parameters. Table 4 shows that the Ca:P ratio in the habitual diet above the median found in the study population was negatively associated with central obesity; the population with a Ca:P ratio above this median had a 64% lower risk (OR: 0.61; 95% CI: 0.41 - 0.92) of central obesity based on WHtR.

**Table 4:** Associations between Ca:P ratio, nutrients, foods and food groups in the habitual diet and global and central obesity of adults of DISANDNT I/JP (2008-2010)

	**BMI **^**1**^			**WC **^**2**^			**WHtR**^**3**^		
		**CI 95%**			**CI 95%**			**CI 95%**	
**All subjects (men and women)**	**OR**	**LL**	**UL**	**OR**	**LL**	**UL**	**OR**	**LL**	**UL**
Ca:P ratio ^4,7^ (mg:mg)	1.19	0.74	1.90	0.74	0.48	1.12	0.61	0.41	0.92
Ca^6,7^ (mg)	1.17	0.74	1.84	0.74	0.49	1.12	0.65	0.43	0.97
P^6,7^ (mg)	1.02	0.65	1.60	0.75	0.50	1.14	0.66	0.44	1.00
Foods and/or food groups^4,5^									
Dairy products (servings)	1.00	0.63	1.58	0.72	0.48	1.08	0.56	0.37	0.84
Whole milk (servings)	0.84	0.53	1.33	0.87	0.58	1.32	0.75	0.50	1.12
Skim milk (servings)	1.91	1.02	3.52	1.3	0.71	2.33	1.43	0.79	2.55
Yogurt (servings)	1.01	0.64	1.59	0.90	0.59	1.35	0.90	0.60	1.35
Cheese (servings)	0.94	0.59	1.49	1.07	0.71	1.63	0.84	0.55	1.27
Meats (servings)	1.11	0.71	1.76	1.21	0.81	1.83	1.29	0.87	1.94
Red meat (servings)	1.00	0.63	1.58	1.11	0.74	1.68	1.02	0.64	1.53
Poultry (servings)	0.95	0.6	1.49	1.33	0.88	2.00	1.29	0.87	1.94
Fish (servings)	0.87	0.55	1.38	1.10	0.73	1.66	0.98	0.66	1.47

^1^ WHO (1998) – global obesity defined as BMI ≥ 30 kg/m^2^; ^2^ NCEP-ATPIII (2002) – central obesity defined as WC > 102 cm for men and > 88 cm for women; ^3^ central obesity defined as WHtR ≥ 0.56 seconds VIEIRA (2009); ^4^ median: Ca:P ratio 0.57, Calcium 485.40 mg, Phosphorus 849.95 mg, Dairy products 0.90 servings, Whole milk 0.32 servings, Skim milk 0.00 servings, Yogurt 0.06 servings, Cheese 0.21 servings, All meats 1.49 servings, Red meat 0.83 servings, Poultry 0.39 servings, Fish 0.14 servings; ^5^ food consumption – servings (BRASIL. Ministério da Saúde. Guia Alimentar para a população brasileira, 2006 [39]/USDA. U.S. Department of Agriculture. Dietary Guidelines for Americans, 2010) [40].

^6^ crude values for calcium and phosphorus.

^7^ Sequential adjustment models by multiple regression - Model 1 – adjusted for age and gender; Model 2 – model 1 plus education level and per capita income; Model 3 - model 2 plus regular physical activity, dairy products and meats intake; Model 4 – model 3 plus calcium for the analysis of phosphorus and phosphorus for the analysis of calcium. After energy-adjusted of nutrients and after sequential adjustment for confounding factors by multiple regression were found positive relations significant between BMI and age, between WC and age and gender (male), and between WHtR and age, and negative between WHtR and education level.

Abbreviations: *Ca:P ratio* Calcium:phosphorus ratio, *OR* Odds ratio, *CI* Confidence interval, *LL* Lower limit, *UL* Upper limit, *BMI* Body mass index, *WC* Waist circumference, *WHtR* Waist-to-height ratio.

Calcium intake above the median was associated with central obesity based on WHtR as a protective factor of 54% (OR: 0.65; 95% CI: 0.43 - 0.97). Among the foods and food groups, the habitual intake of dairy products above the median (0.9 servings, corresponding to 180 ml of milk or yogurt or 36 g of cheese) was also a protective factor for central obesity based on a WHtR of 79% (OR: 0.56; 95% CI: 0.37 - 0.84) whereas the consumption of skim milk above the median was positively associated with global obesity (BMI) (OR: 1.91; 95% CI: 1.02 - 3.52).

## Discussion

These results show that a Ca:P ratio above 0.57 in the habitual diets of subjects was not associated with global obesity (BMI) but was negatively associated with central obesity based on WHtR, which is considered risk factor for cardiovascular diseases [32,33]. Habitual dietary calcium intake above the median value (485.4 mg) was negatively associated with central obesity based on WHtR becoming a protective factor. The same association occurred regarding the consumption of dairy products, although with a greater protective effect.

No study relating the Ca:P ratio in the habitual diet with obesity was found in the literature. However, a higher dietary calcium intake has been identified by some researchers as a protective factor for overweight [6,7,42-44]. The consumption of phosphorus, in addition to being associated with lower calcium absorption [5,45,46], was positively associated with global obesity and central obesity based on WC in the study by Beydoun et al [8]. We should mention that this was the only study found that related phosphorus intake to obesity. The Ca:P ratio median in this study was used as the cutoff point, from which a protective effect for central obesity was observed, and this median was much smaller than the value suggested in the literature for bone health promotion [15,21].

In the above study [8], which reported calcium intake as a protective factor and phosphorus as a risk factor for obesity, the mean Ca:P ratio in the habitual diet was 0.65. This average value was calculated by us from data on average calcium and phosphorus intake measured during the study; however, this ratio was not discussed in that study as a factor that affects obesity.

Kemi et al. [15] studied the effects of this ratio on calcium metabolism and serum parathyroid hormone and also found values lower than those suggested [15,21] (average 0.74). They also observed that values below 0.65 were deleterious whereas values above 0.65 were not. Although this study focused on bone health in a population with calcium intake that was sufficient or above the requirements, the ratio found in both studies was well below that suggested in the literature, leading us to believe that even low Ca:P ratios have a beneficial effect. This beneficial effect was observed in both the bone health study and the present study on obesity. It is noteworthy that among the subjects we studied, none had a Ca:P ratio greater than or equal to the value suggested in the literature of 1.3:1.0 [15,21].

With regard to calcium, approximately 84% of the subjects did not meet the requirements for this nutrient. A higher proportion (99%) was found in a study conducted in Brazil with individuals over 40 years of age [12]. Several researchers have also found insufficient intake of this mineral in other countries [8,10,47]. In a study assessing the calcium intake of a population over 2 years of age, it was found that only 32.30% of subjects showed adequate intake of this nutrient. In the adult population, a similar proportion was found: 38.80% for the population aged 19 to 30 years and 33% for the population aged 31 to 50 years [10].

It is important to note that the DRIs used in this study are lower than those used in the previously mentioned studies because of recent updates in the requirements for this nutrient, thereby reducing the percentage of individuals who did not meet these needs in relation to previous studies.

The opposite situation has been found for phosphorus intake, which is 2 to 3 times higher than required [14,15]. Beydoun et al. [8] associated the consumption of dairy products and nutrients with obesity, central obesity and metabolic syndrome and found that, for each daily intake of 100 mg of phosphorus, the prevalence of global obesity (BMI) and central obesity (WC) increased by 7% and 6%, respectively. In the present study, average phosphorus intake was 1.6 times greater than the requirements, and the highest daily amount ingested was 5 times higher than the requirements. However, despite its high consumption, phosphorus was not related to any obesity diagnostic parameter, suggesting that the protective effect achieved by the high Ca:P ratio (which could be achieved by a higher calcium intake and lower phosphorus intake) was because calcium alone was protective for central obesity based on WHtR. A similar relationship occurred between dairy consumption and central obesity based on WHtR, which corroborates the above discussion because this food group is not only a good source of phosphorus but also of calcium, unlike many other foods that contain large amounts of phosphorus but low amounts of calcium.

Most researchers who have linked the consumption of calcium and dairy products with central obesity based on waist circumference, which is the most widely used parameter, found an inverse relationship [8,43,48-50]; however, in the present study and the study by Brooks et al. [51] this relationship was not found.

This study also investigated the association between food intake variables and the waist-to-height ratio as a parameter for diagnosing central obesity and found a relationship between Ca:P ratio in the habitual diet, calcium intake and dairy products. Although the WHtR is considered a parameter for diagnosing central obesity, which when present represents a risk factor for cardiovascular diseases [32,33,52,53], no studies were found in the literature relating this parameter with the intake variables mentioned. This finding is another new piece of evidence provided by this study.

The consumption of skim milk was positively associated with global obesity. However, the vast majority of the study population did not consume this food, resulting in a consumption median equal to zero, which prevented further statistical analyses. Studies with a larger number of consumers should be performed.

This study has the following limitations: the absence of information on comorbidities of individuals and its cross-sectional design, which does not allow us to establish a cause-and-effect relationship. However, this type of study model determines risk factors and identifies previously unknown relationships in groups or in the general population. Other limiting factor was the exclusion of 17.67% of the sample for dietary analyses due to incomplete data. Finally, the individuals studied did not reach a Ca:P ratio greater than or equal to values suggested in the literature, thus preventing a comparative analysis with individuals who have met these recommendations.

Regarding its strengths, this study has a stratified and systematic sampling design and uses recent data, allowing the representation of the adult study population. This study also used a validated handbook for the study population, with measures of food prepared at home, thus facilitating a more accurate quantification of portion sizes consumed. Another positive factor was the coordination of home visits by master’s students and weekly meetings of all staff with the research coordinators throughout the training and data collection periods, facilitating the standardization of the methodology used throughout the study.

## Conclusions

In conclusion, an above median Ca:P ratio in the habitual diet was negatively associated with central obesity based on WHtR, although most individuals had low calcium intake and high phosphorus intake. Moreover, the habitual consumption of calcium and dairy above the median value was related as a protective factor to central obesity based on WHtR. Experimental and cross-sectional studies should be performed with animals and with individuals considered "healthy", eutrophic and with good bone health to elucidate whether a good Ca:P ratio for bone health also prevents obesity, thus supporting the use of this ratio as part of a balanced diet and as one more nutritional strategy against obesity.

## Abbreviations

BMI: Body mass index; BMD: Bone mineral density; Ca: Calcium; CI: Confidence interval; DISANDNT I/JP: First diagnosis and intervention of food and nutritional status and the most prevalent non-communicable diseases of the population from the city of João Pessoa-PB/Northeastern Brazil"; DRI: Dietary reference intake; EAR: Estimated average requirement; FQQFC: Frequency questionnaire quantitative food consumption; LL: Lower limit; OR: Odds ratio; P: Phosphorus; POF: Pesquisa de Orçamentos Familiares (Household Budget Survey); SD: Standard deviation; UL: Upper limit; WC: Waist circumference; WHtR: Waist-to-height ratio

## Competing interest

The authors declare that they have no competing interests.

## Authors’ contributions

DCP contributed to data management, statistical analyses, data interpretation and manuscript writing. RPAL contributed to data acquisition, data management and data interpretation. RTL contributed to the study design and coordinated the data collection. MCRG, LCSLM, and SCCF have been involved in drafting the manuscript or revising it critically for important intellectual content. RMM contributed to the study design and the statistical analyses. LSRA contributed to manuscript writing and have been involved in drafting the manuscript or revising it critically for important intellectual content. MJCC contributed to the study design, statistical analyses, data interpretation, manuscript writing, coordinated the data collection and have been involved in drafting the manuscript or revising it critically for important intellectual content. All authors read and approved the final manuscript.
